# MicroRNA-Based Separation of Cortico-Fugal Projection Neuron-Like Cells Derived From Embryonic Stem Cells

**DOI:** 10.3389/fnins.2019.01141

**Published:** 2019-10-23

**Authors:** Tadashi Sunohara, Asuka Morizane, Satoshi Matsuura, Susumu Miyamoto, Hirohide Saito, Jun Takahashi

**Affiliations:** ^1^Department of Clinical Application, Center for iPS Cell Research and Application, Kyoto University, Kyoto, Japan; ^2^Department of Neurosurgery, Kyoto University Graduate School of Medicine, Kyoto, Japan; ^3^Department of Life Science Frontiers, Center for iPS Cell Research and Application, Kyoto University, Kyoto, Japan

**Keywords:** corticofugal projection neuron, microRNA-responsive mRNA switch, miRNA-124-3p, embryonic stem cell derived-neuron, cell sorting

## Abstract

The purification of pluripotent stem cell-derived cortico-fugal projection neurons (PSC-CFuPNs) is useful for disease modeling and cell therapies related to the dysfunction of cortical motor neurons, such as amyotrophic lateral sclerosis (ALS) or stroke. However, no CFuPN-specific surface markers for the purification are known. Recently, microRNAs (miRNAs) have been reported as alternatives to surface markers. Here, we investigated this possibility by applying the miRNA switch, an mRNA technology, to enrich PSC-CFuPNs. An array study of miRNAs in mouse fetal brain tissue revealed that CFuPNs highly express miRNA-124-3p at E14.5 and E16.5. In response, we designed a miRNA switched that responds to miRNA-124-3p and applied it to mouse embryonic stem cell (ESC)-derived cortical neurons. Flow cytometry and quantitative polymerase chain reaction (qPCR) analyses showed the miRNA-124-3p switch enriched CFuPN-like cells from this population. Immunocytechemical analysis confirmed vGlut1/Emx1/Bcl11b triple positive CFuPN-like cells were increased from 6.5 to 42%. Thus, our miRNA-124-3p switch can uniquely enrich live CFuPN-like cells from mouse ESC-derived cortical neurons.

## Introduction

Corticofugal projection neurons (CFuPNs) are projection neurons that connect the cerebral cortex and subcortex and transmit excitatory input to the subcerebral nucleus. CFuPNs compose the corticothalamic tract, corticostriatal tract, corticorubral tract, corticotectal tract, corticobulbar tract, and corticospinal tract, and are crucial players in sensorimotor systems (Molnár and Cheung, 2006; Leone et al., 2009; Greig et al., 2013; Jose et al., 2015). In some neurodegenerative disorders such as amyotrophic lateral sclerosis (ALS) and primary lateral sclerosis (PLS), corticospinal motor neurons (CSMNs), which are a subset of CFuPNs, degenerate. In other diseases like stroke, damage to CFuPNs is associated with a decline in quality of life.

Several groups have used CFuPNs to model these disorders *in vitro* (Sances et al., 2016) and to develop cell therapies to treat them (Gaillard et al., 2007; Steinbeck et al., 2012; Espuny-Camacho et al., 2013; Motono et al., 2016; Sano et al., 2017). Because primary CFuPNs are difficult to procure, these works have relied on differentiating pluripotent stem cells (PSCs) to the CFuPN fate (Zhu et al., 2016), but the resulting cell populations are often heterogeneous. Typically, cell sorting is done after the differentiation to purify the desired cell population (Okano et al., 2013; Doi et al., 2014; Takeda et al., 2018), but this is not an option for CFuPNs, because no specific cell surface markers or antibodies are known. The insertion of a reporter gene is one option for the purification, but this approach prohibits cell therapies.

We have developed a microRNA (miRNA)-responsive modified mRNA system (the miRNA switch) that post-transcriptionally regulates transgene expressions in response to the expression of specific and arbitrary miRNAs in a cell (Miki et al., 2015; Endo et al., 2016; Parr et al., 2016). This feature permits an alternative to surface markers for cell purification. Indeed, cell purifications using miRNA switches have been reported for cardiomyocytes, endothelial cells, hepatocytes, insulin-producing cells (Miki et al., 2015), and undifferentiated human PSCs (Parr et al., 2016) from PSC-derived heterogenous cell populations.

The present study applied the miRNA switch to enrich CFuPNs similarly. Because the miRNA expression profile of CFuPNs is not well known, we first clarified the miRNA profile by a microarray analysis of sorted primary CFuPNs acquired from mouse embryonic brain. We further examined mouse embryonic stem cell (ESC)-derived coritcal cells with miRNA switches to detect candidate miRNA targets in the CFuPNs.

## Materials and Methods

### Establishing Bcl11b-IRES-EGFP Knock-in Mice

Bcl11b + CFuPNs were purified from Bcl11b-IRES-EGFP knock-in mice. We established a targeting vector using Red/ET BAC recombination technology (Gene Bridges). The mouse BAC clone, RP23-351K8, containing the mouse Bcl11b gene locus was purchased from BACPAC Resources. Four steps were required for the recombination (Supplementary Figure S1). First, the IRES-EGFP reporter cassette, IRES-EGFP-polyA-rox-PGK-EM7-Bsd-polyA-rox, was targeted into RP23-351K8 with the homology arm of both ends of the cassette to create IRES-EGFP knock-in BAC. Second, the IRES-EGFP reporter cassette with the homology arm of the Bcl11b sequence was retrieved from IRES-EGFP knock-in BAC to create the targeting vector. 20 μg linearized targeting vector was transfected into mouse ESCs (v6.5) by electroporation followed by cultivation with Blasticidin S (15 μg/mL) for 7 days. After picking up the surviving colonies, Dre-recombinase (Gene Bridges) was transfected into the surviving colonies by electroporation. The loss of Blasticidin S resistance was then examined in single cells from the colonies. Mouse ESCs with a loss of Blasticidin S resistance were termed Bcl11b-IRES-EGFP knock-in mouse ESCs. To establish Bcl11b-IRES-EGFP knock-in mice, Bcl11b-IRES-EGFP-knock-in mouse ESCs were trypsinized, and 7–8 cells per embryo were injected into the blastocoels of E3.5 mouse blastocysts. Injected blastocysts were then transferred into the uteri of pseudo-pregnant ICR mice at E2.5. Chimeric mice were confirmed by genotyping and used as ICR-v6.5/Bcl11b-IRES-EGFP. After obtaining chimeric mice, F1 mice genotyped for EGFP were used as studs to establish the Bcl11b-IRES-EGFP knock-in mouse line. F2 mice were used as Bcl11b-IRES-EGFP knock-in mice after confirmation of GFP emission by fluorescence stereoscopic microscopy.

### Cortical Cell Harvesting and Cell Sorting

**FIGURE 1:**
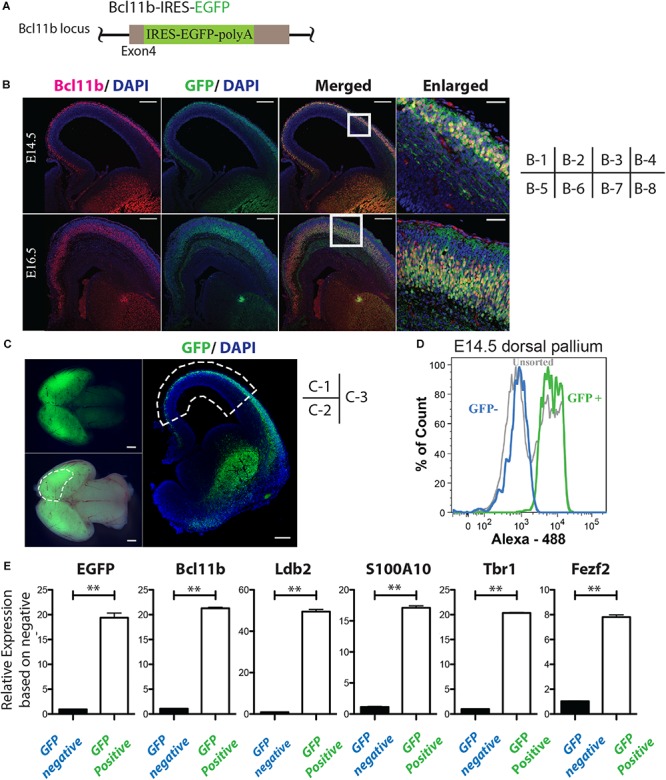
Isolation of Bcl11b-positive cells from mouse embryonic brain cortex. **(A)** Schematic of the IRES-EGFP knock-in Bcl11b locus. **(B)** Co-expression of GFP and Bcl11b in the cortical plate of E14.5 and E16.5 mouse embryo by immunohistochemical analysis. Bcl11b (red), GFP (green), DAPI (blue). Scale bars, 200 μm (B-1, -2, -3, -5, 6, -7) and 50 μm (B-4, -8). B-4 and B-8 are magnified images of the squared area in B-3 and B7, respectively. **(C)** Sampling of the dorsal pallium from mouse embryo (E14.5). The manually dissected dorsal pallium is circled by the dashed lines in C-2 and C-3. Fluorescence stereoscopic microscopy images (fluorescence: C-2, merged: C-3). Immunohistochemical image of the coronal section of the sampled brain (C-3). GFP (green), DAPI (blue). Scale bars, 100 μm (C-1, 2) and 200 μm (C-3). **(D)** Separation of CFuPNs by GFP expression with FACS from the sampled tissue in panel **(C)**. **(E)** qPCR analysis of the separated cells. The expression of CFuPN-specific genes was increased in the GFP-positive population. Student’s *t*-test. ^∗∗^*p* < 0.0001. *n* = 3.

Cerebral cortices (i.e., the dorsal pallium) were dissected manually under a microscope from E14.5 and E16.5 Bcl11b-IRES-EGFP knock-in mice (Figure 1C). Harvested cortices were gently dissociated into single cell suspensions by Neuron Dissociation Solution S (Wako, Japan) and resuspended in fluorescence activated cell sorting (FACS) Buffer [phenol-free, Ca^2+^Mg^2+^-free Hank’s balanced salt solution (HBSS; Invitrogen, Waltham, MA, United States)] containing 2% FBS (HyClone, United States), 20 mM D-glucose (Wako), and 50 mg/ml penicillin/streptomycin (Invitrogen). Samples were filtered through cell-strainer caps (35 μm mesh; BD Biosciences, Franklin Lakes, NJ, United States) into FACS Buffer. The cells were analyzed and sorted by a FACS AriaIII cell sorter and FACSDiva software (BD Biosciences). A 100 μm ceramic nozzle (BD Biosciences) with a sheath pressure of 20–25 psi and an acquisition rate of 1500–3000 events/s was used for the sorting. Gates were set as follows. A positive gate was set so that less than 0.1% of events exceeded the threshold in GFP-negative population samples, and a negative gate was set so that less than 0.1% of GFP-positive cells were included in the analysis of the GFP-negative sorted cells (Figure 1D). Sorted cells were collected in Nerve Cell Culture Medium (DS Pharma Biomedical, Japan) and centrifuged for RNA extraction.

### RNA Extraction and Quality Control for Microarray

Cells were lysed using QIAzol lysis reagent (Qiagen), and total RNA was purified using the miRNeasy Mini Kit (Qiagen) according to the manufacturer’s protocol. The RNA concentration and purity were determined by measuring the A260/280 ratio using Nanodrop (Thermo Fisher Scientific). For microarrays, confirmation of the RNA quality was performed using a 2100 Bioanalyzer Instrument and Agilent RNA 6000 Nano Assay (Agilent Technologies). The RNA integrity number of all samples was over 9.8.

### miRNA Microarray Analysis

miRNA expression profiling was performed using the GeneChip miRNA4.0 Array and Flashtag Bundle (Affymetrix) according to the manufacturers’ protocols. Biotinylated cRNA were prepared according to the standard Affymetrix protocol from 500ng/8uL total RNA (Expression Analysis Technical Manual, miRNA 4.0 ARRAY & FLASHTAGTM BUNDLES, Affymetrix). GeneChips were scanned using the Affymetrics GeneChip Scanner 3000. The data were analyzed with Microarray Suite version 5.0 (MAS 5.0) using Affymetrix default analysis settings and global scaling as normalization method. The trimmed mean target intensity of each array was arbitrarily set to 100. Then further data analysis was done with GeneSpring GX 13.1 software (Agilent Technologies).

### miRNA Switches

miRNA-responsive mRNAs (miRNA switches) were encoded on modified mRNA (Miki et al., 2015; Endo et al., 2016) to post-transcriptionally regulate a fluorescent reporter (blue fluorescent protein, BFP) in response to the activity of a miRNA expressed in living cells (see schematic in Figure 3). miRNA switches were generated using a MEGAScript T7 kit (Ambion) and a modified protocol (Matsuura et al., 2018; Warren et al., 2013) In the reaction, pseudouridine-5′-triphosphate and 5-methylcytidine-5′-triphosphate (TriLink BioTechnologies) were used instead of uridine triphosphate and cytosine triphosphate, respectively. Guanosine-5′-triphosphate was 5-fold diluted with Anti-Reverse Cap Analog (New England Biolabs) before the IVT reaction. Reaction mixtures were incubated at 37°C for 4 hr, mixed with TURBO DNase (Ambion), and further incubated at 37°C for 30 min. The resulting mRNAs were purified using a FavorPrep Blood/Cultured Cells total RNA extraction column (Favorgen Biotech), incubated with Antarctic Phosphatase (New England Biolabs) at 37°C for 30 min, and then purified again using an RNeasy MinElute Cleanup Kit (Qiagen).

The reporter was translationally repressed when the mature target miRNA binds to its completely complementary sequence in the miRNA switch.

### Cell Analysis Using miRNA Switches

The transfection of miRNA switches was performed as previously described (Miki et al., 2015) with minor modifications. Briefly, reverse transfection was done with Lipofectamine MessengerMAX Transfection Reagent (Thermo Fisher Scientific, United States) following the manufacturer’s protocol. 20 ng/well of miRNA-switches with tagBFP and the mRNA of hmAG or iRFP (control) were transfected into mouse ESC-derived cortical neurons that were enzymatically dissociated into single cells and replated onto 96 PDL-coated wells or a PDL-coated 6-well flat bottom plate. After 24 h, the cells were collected and analyzed with BD AriaIII.

### Immunohistochemistry

For *in vivo* studies, each embryo was gently and promptly dissected to take out brains in iced HBSS. The brains were fixed in 4% paraformaldehyde for 1 day, transferred to 15% sucrose-containing PBS for 1 day and then to 30% sucrose-containing PBS at 4°C until use. Each brain was embedded with O.C.T. compound (Sakura Finetek) and cryosectioned into 20 μm sections using Cryostat (CM3050 S; Leica Biosystems). Double- or triple-labeled immunohistochemical analysis was carried out after permeabilization and blocking with 0.3% TritonX-100 and 2% skim milk. The immuno-reactive cells were visualized using a fluorescence microscope (BZ-X710; Keyence, Osaka, Japan) and a confocal laser microscope (LSM710, Carl Zeiss Inc., Germany). The primary antibodies used are as follows:

anti-GFP (1:1000, MBL international Cat#598 PRID: AB_591819).

anti-Ctip2[25B6] ChIP Grade (1:500, Abcam, Cat#ab18465 PRID: AB_2064130).

anti-Bf1 (1:1000, Takara, Cat#M227).

anti-Laminin (Abcam, ab11575) (1:500, Abcam Cat#ab11575 PRID: AB_298179).

anti-Tuj1 (Covance, MMS435p) (1:2000, Covance Cat#MMS435p PRID: AB_2313773).

anti-Emx1 (1:500, Takara, Cat#M196).

anti-Lhx2 (1:100, Santa Cruz, Cat#sc19342 PRID: AB_2135663).

anti-vGlut1 (1:2000, Synaptic Systems, Cat#135 303 PRID: AB_887875).

### Reverse Transcription and Quantitative PCR

To detect the mRNA transcripts, purified RNA was reverse transcribed using the SuperScriptIII First-Strand Synthesis System (Invitrogen). Quantitative PCR reactions were carried out with Power SYBR (Applied Biosystems) according to the manufacturer’s instructions. The expression level of each gene was normalized to that of GAPDH using the ΔΔ-Ct method. The primer sequences used are shown in Table 1.

**TABLE 1:** Primer sequence for qPCR.

**Oligo sequence (5′–3′)**	**Oligo name**
CCGCCTGGAGAAACCTGCCAAGT	mGAPDH_Fw
GGGAGTTGCTGTTGAAGTCGCAGG	mGAPDH_Rv
GCCCAGGTTTCGACAGACT	mu_S100a10_Fw
CCACTAGTGATAGAAAGCTCTGGA	mu_S100a10_Rv
AGGAGAGTATCTGAGCCAGTG	mBcl11b_Fw
GTTGTGCAAATGTAGCTGGAAG	mBcl11b_Rv
GAGGAAAGAGAAAGGAAGACTAGG	mSatb2_Fw
CCATGGACAGAGCCCCAGCC	mSatb2_Rv
TATAACCTCACCCGCCACAT	m_fezf2_Fw
CACAAAACTCGCAGACGAAG	m_fezf2_Rv
ACCCTGCCCTGTGAGTCTTT	Foxg1_Fw
GACCCCTGATTTTGATGTGTG	Foxg1_Rv
GCCACTGCTTACTCGCACCT	Reelin_Fw
GCCACACTGCTCTCCCATCT	Reelin_Rv
TCCTGGAACAAGCCAAGAGG	Cux1_Fw
CTGTAGGATGGAGCGGATGG	Cux1_Rv
CATCGGCATCAAACGGAGA	Tbr1_Fw
CGCCAAAATCACATCCACAA	Tbr1_Rv
GGCAAGCTGACCCTGAAGTT	EGFP_Fw
TTCTCGTTGGGGTCTTTGCT	EGFP_Rv

*Fw: forward; Rv: reverse.*

### Statistical Analysis

Statistical analyses were performed using GraphPad Prism 5 (GraphPad Prism software, CA, United States). Data from sorted samples were compared by *t*-tests (Figure 1E and Supplementary Figures S2C, S4A,B). Data are presented as means ± SD.

### ESC Culture and Induction of Mouse ESC-Derived Cortical Neurons

Bcl11b-IRES-EGFP knock-in mouse ESCs were maintained and cultured as previously reported (Watanabe et al., 2005). The differentiation medium was constituted of G-MEM supplemented with 10% Knockout Serum Replacement (KSR; Invitrogen), 2 mM L-glutamine (Thermo Fisher Scientific), 1 mM Sodium pyruvate solution (SIGMA), 0.1 mM MEM Non-Essential Amino Acids Solution (Thermo Fisher Scientific), 0.1 mM 2-Mercaptoethanol (Wako, Japan), 10 μM SB431542 (Merck), and 20 nM Wnt-C59 (Cellagen Technology) (Eiraku et al., 2008; Motono et al., 2016). For the SFEBq culture, ESCs were dissociated into single cells in 0.05% trypsin-EDTA (Invitrogen) and quickly reaggregated in the differentiation medium (4000 cells/150 μl/well) using Prime Surface 96U plates (Sumilon). To induce mouse ESC-derived cortical neurons, day 6 cell aggregates were transferred to a 10 cm bacterial-grade dish in N2 medium (DMEM/F12 supplemented with N2, B27, 0.1 mM 2-ME, and 2 mM glutamine) supplemented with 50 ng/ml FGF8b (R&D systems) and 5 μM cyclopamine (Enzo life sciences) for dorso-anteriorization of the telencephalon.

## Results

### Purification of Cortical Bcl11b+ Corticofugal Projection Neurons From Mouse Embryo

To investigate which miRNAs are up-regulated in CFuPNs, we established Bcl11b-IRES-EGFP knock-in mice (Figure 1A and Supplementary Figure S1). The brains of Bcl11b-IRES-EGFP knock-in mice were sampled at E14.5 and E16.5. Coronal sections of the brains showed the co-expression of GFP and Bcl11b in the cortical plate of the cerebral cortex (Figure 1B).

**FIGURE 2:**
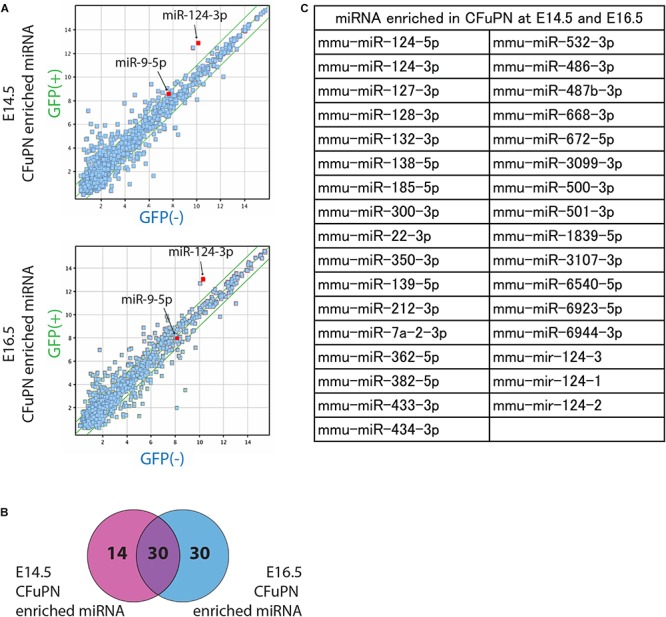
miRNA profiles enriched in CFuPNs at E14.5 and E16.5. **(A)** Scatterplots of the microarray analysis comparing GFP+ versus GFP- populations on E14.5 and E16.5. Green lines indicate fold change ±2.0. The locations of miRNA-124-3p and miRNA-9-5p are indicated. **(B)** Venn diagram of the microarray analysis showing 44 miRNAs were enriched in the GFP+ population at E14.5 and 60 at E16.5. 30 miRNAs were enriched at both times. **(C)** List of miRNAs enriched in the GFP+ population at both E14.5 and E16.5. miRNA-124 derivatives are included in the list [-3p, -5p, and stem-loop (124-1, 124-2, and 124-3)].

**FIGURE 3:**
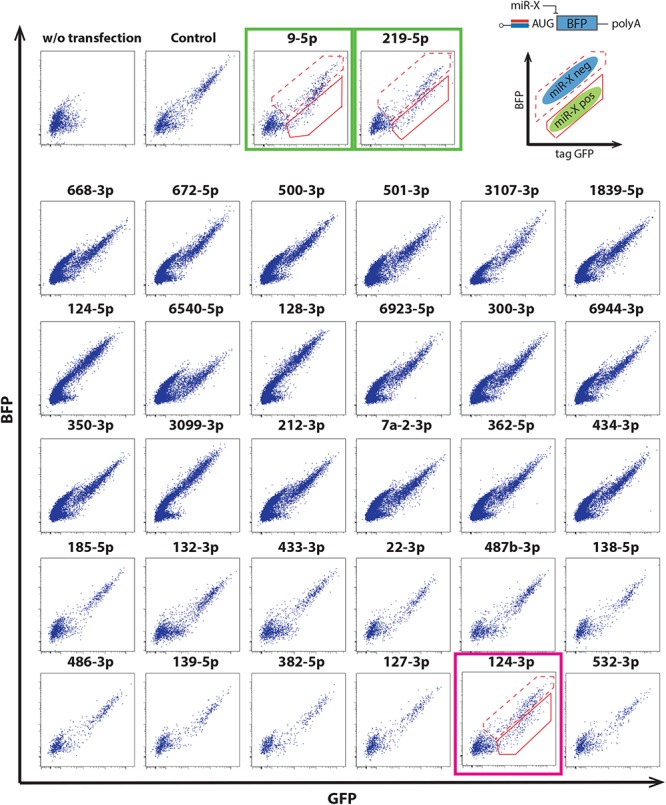
Application of miRNA switches using the 30 mRNAs in Figure 2C to mouse ESC-derived cortical neurons at differentiation day 15. miRNA-124-3p switch separated mouse ESC-derived cortical neurons into two populations (magenta box). miRNA-9-5p and miRNA-219-5p switches also separated the cells into two populations (green boxes). Modified RNAs that encoded miRNA switches respond to the activity of the target miRNA when transcribed into living cells (upper right). The solid and dotted outlines in the dot plots correspond to the miRNA-negative and -positive fractions, respectively. Translational repression of the reporter protein (BFP) occurs when mature miRNA binds to a completely complementary sequence that is optimally placed within the 5′-UTR of the miRNA switch.

GFP emission was confirmed in the brains by fluorescence stereoscopic microscopy (Figure 1C). The dorsal pallium was dissected at E14.5 and E16.5 (Figure 1C-2,C-3), and flow cytometry analysis showed clear separation of the GFP-positive and -negative populations (Figure 1D and Supplementary Figure S2A). The percentage of GFP-positive and -negative sorted cells of the total population at E14.5 was 36.6 ± 1.72 and 41.9 ± 0.81%, and at E16.5 it was 24.0 ± 6.58 and 23.1 ± 3.13%, respectively (Supplementary Figure S2B). A quantitative polymerase chain reaction (qPCR) analysis of each population showed markers of CFuPN (Bcl11b, Ldb2, Tbr1, Fezf2, etc.) were upregulated in the GFP-positive fraction (Figure 1E and Supplementary Figure S2C; *p* < 0.0001, Student’s *t*-test). Samples from both populations were collected for the following microarray analysis.

### miRNA Profiles Enriched in CFuPN

We performed a miRNA microarray with the samples sorted from mouse embryo to screen for miRNAs enriched in the GFP-positive CFuPN fraction. We found 44 miRNAs and 60 miRNAs were up-regulated at E14.5 and E16.5, respectively (Figure 2A), and 30 miRNAs were specifically enriched in CFuPNs (Figure 2B). Interestingly, miRNA-124, which is one of the most frequently reported miRNAs in neurons, was highly expressed and strongly enriched in the GFP-positive CFuPN fraction. Additionally, the 5p- and 3p-ends (miRNA-124-5p and miRNA-124-3p) and the stem-loop of miRNA-124 (mir-124-1, mir-124-2, mir-124-3) were also enriched in the GFP-positive fraction (Figure 2C).

### miRNA Switch Activity in Mouse ESC-Derived Cortical Neurons

As a next step, we applied miRNA switches deduced from the microarray analysis to mouse ESC-derived cortical neurons (Eiraku et al., 2008; Motono et al., 2016). The induced neurons formed as a dorsal pallium-oriented spheroid at differentiation day 15. To induce CFuPNs phenotype, the cells were treated with cyclopamine, a dorsalization factor and FGF8b, an anterization factor, from differential day 6–15 (Supplementary Figure S3A). In the spheroid, Foxg1 and Lhx2 double positive telencephalic rosettes were detected (Supplementary Figures S3B,E). These rosettes were surrounded by Bcl11b-positive neurons (Supplementary Figures S3C,F), which were positive for TuJ1, a mature neuronal marker (Supplementary Figure S3D), and for Emx1 (Supplementary Figure S3G), a cortical plate marker. Rosettes were lined with Laminin on the outer side (Supplementary Figure S3D). Finally, qPCR revealed that the telencephalic markers and cortical markers gradually increased with differentiation (Supplementary Figure S3H).

We thus evaluated miRNA switch activity in mouse ESC-derived cortical neurons. Along with miRNA-124-3p, the switches were designed to respond to several miRNA, including miRNA-9-5p and miRNA-219-5p, which are reported to be active in neural development (Dugas et al., 2010; Bonev et al., 2012; Li et al., 2016). miRNA-124-3p, 9-5p and 219-5p switches separated the induced neurons into two populations (Figure 3).

### Separation of ESC-Derived Cortical Neurons by miRNA Switches

**FIGURE 4:**
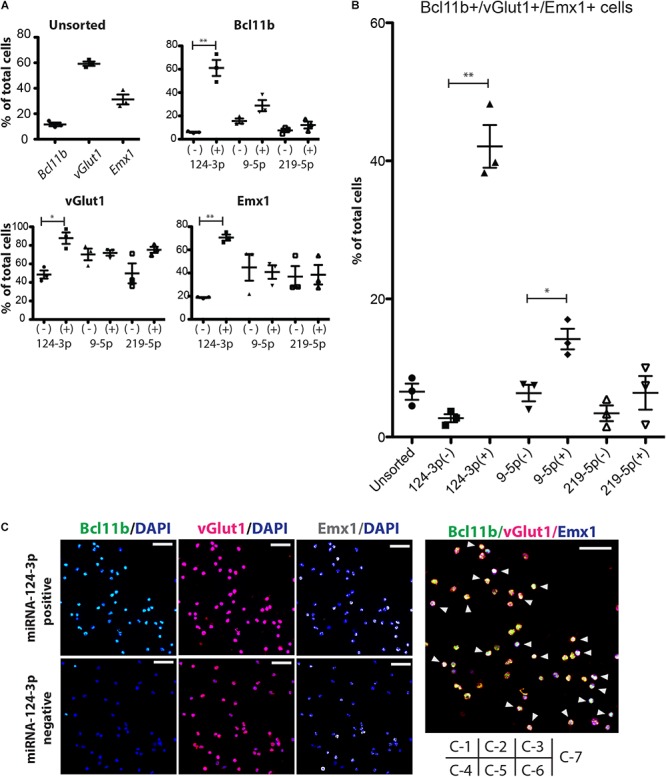
Enrichment of CFuPN-like cells by miRNA switches. **(A)** Immunocytochemistry results for the CFuPN markers Bcl11b, vGlut1 and Emx1. miRNA-124-3p switch had the highest separation efficiency. **(B)** Quantitative assessment of BcL11b, vGlut1 and Emx1 triple positive cells. Student’s *t*-test. ^∗^*p* < 0.05, ^∗∗^*p* < 0.001. *n* = 3. **(C)** Representative immunocytochemical images of the cells separated by the miRNA-124-3p switch. Bcl11b (green), DAPI (blue), vGlut1 (red), and Emx1. Scale bars, 50 μm (white in C-3, -6) and 20 μm (blue in C-7). Arrowheads in C-7: triple positive CFuPN-like cells.

As a next step, we sorted the ESC-derived cortical neurons by FACS and quantified the separation efficacy. In this experiment, BCl11b-EGFP knock-in mouse ESCs were differentiated to cortical neurons, in which the CFuPNs expressed GFP. The percentage of GFP-positive cells before separation was 8.96 ± 1.75%, and the percentage of Bcl11b+ cells was 11.6 ± 2.51% by immunocytochemistry (Figure 4A, unsorted). We next transfected the cells with tagBFP-coding miRNA switches and iRFP control mRNAs. Switch activation in the cells was detected by a lower expression level of transfected BFP (Supplementary Figure S4A). Following the transfection, we found the percentage GFP-positive cells increased to 63.00 ± 6.86% by miRNA-124-3p, 41.80 ± 17.55% by miRNA9-5p, and 33.20 ± 5.92% by miRNA219-5p (Supplementary Figure S4B).

Next, we evaluated the sorted cells by immunocytochemistry. The miRNA switch activity showed that CFuPNs in early development express Bcl11b, Emx1, and vGlut1 (Figure 4A). Specifically, miRNA-124-3p switch enriched vGlut1-positive, Emx1-positive and Bcl11b-positive cells. Quantitative analysis revealed that 42.11 ± 5.36% of the cells in the miRNA-124-3p positive fraction were triple positive for Bcl11b, Emx1, and vGlut1, indicating the CFuPN phenotype (Figures 4B,C). On the other hand, the ratio of triple positive cells in the unsorted population was 6.58 ± 0.61% (Figures 4B,C). These results indicated that CFuPN-like cells were enriched 6.5-fold by the miRNA-124-3p switch. The miRNA-9-5p switch could also separate triple positive cells, but to a lesser degree (14.18 ± 2.59%, Figure 4B). The miRNA-219-5p switch did not significantly separate CFuPN-like cells (Figure 4B). Finally qPCR analysis confirmed the miRNA-124-3p switch positive fraction had a significantly higher level of Bcl11b and Ldb2 than the negative fraction, but that the percentage of Satb2, Tbr1 and S100a10 were the same (Supplementary Figure S5). This means that miRNA-124-3p switch could enrich subcerebral projection neuron as a subtype of CFuPN (Molyneaux et al., 2015).

## Discussion

In this study, we profiled the miRNA expressions of CFuPNs in the course of corticogenesis and investigated three miRNA switches for the separation of CFuPNs. As a result, we found that miRNA 124-3p is a specific marker of CFuPNs.

Several diseases or trauma related to the brain, such as ALS and stroke, lead to the degeneration or death of cortical neurons. Based on neurodevelopmental studies, scientists have theorized that the transplantation of CFuPNs is a promising regenerative medicine related to these ailments. Because primary CFuPNs are extremely difficult to procure, scientists have turned to PSCs and inducing these cells to differentiate into CFuPNs. A major challenge in translating these differentiation protocols to clinical therapy, however, is the heterogeneous population in which the CFuPNs. This problem is not exclusive to CFuPN differentiation, and most cell therapies have an added step in which the desired cell type is purified with a collection of surface markers. However, such surface markers are unknown for CFuPNs (Yoneshima et al., 2006; Azim et al., 2009; Leone et al., 2009; Shi et al., 2012; Greig et al., 2013; Jose et al., 2015; Molyneaux et al., 2015; Oishi et al., 2016).

As an alternative, we have investigated miRNA as an intracellular marker of cell types. There are over 2000 known miRNA expressed in humans (Hammond, 2016). miRNAs are small non-coding RNAs of about 22 nucleotides in size (Lee, 1993). They function to silence RNA and regulate genes post-transcriptionally (Ambros, 2004; Bartel, 2004). Studies have demonstrated that differential miRNA expression patterns can not only mark different cell types (Landgraf et al., 2007), but even disease progression (Jones et al., 2014). Indeed, miRNAs is indispensable for structured layer formation of corticogenesis in development (Makeyev et al., 2007; Shibata et al., 2008; Zhao et al., 2009; Nowakowski et al., 2011; Hong et al., 2013; Nowakowski, 2013; Pollock et al., 2014; Davis et al., 2015; Santa-Maria et al., 2015; Stappert et al., 2015; Roese-Koerner et al., 2016; Xue et al., 2016), and have a crucial role in neuronal differentiation and subtype specification (Yoo et al., 2009; Gaughwin et al., 2011; He et al., 2012; Anderegg et al., 2013; Cremisi, 2013; Stappert et al., 2013, 2015; Pollock et al., 2014; Zhao et al., 2015). MicroRNA also have a important role in cell-fate determination (Szulwach et al., 2010; Coolen et al., 2012; Xue et al., 2013; Taylor et al., 2014). We have reported the miRNA switch, synthetic mRNA that controls the expression of an arbitrary gene based on the expression level of an arbitrary miRNA. In the current study, we show that preparing a miRNA switch that includes a completely complementary sequence for miRNA-124-3p and mRNA for the expression of fluorescent protein, we could purify CFuPNs at unprecedented efficiency.

To identify miRNA-124-3p as an intracellular marker of CFuPNs, we first examined miRNA expression levels by analyzing cells that expressed a set of transcription factors. CFuPNs are characterized by the expression of Bcl11b (Ctip2), Fezf2, Tbr1, vGlut 1, and Emx1. The expression of these factors are considered the most reliable markers of CFuPNs. However, to detect their expression requires genomic modification of the cells, thus prohibiting the cells from clinical therapies. On the other hand, miRNA switches do not integrate into the genome and have a short half-life in the cell, making them one of the safer biotechnologies available for cell therapies.

**FIGURE 5:**
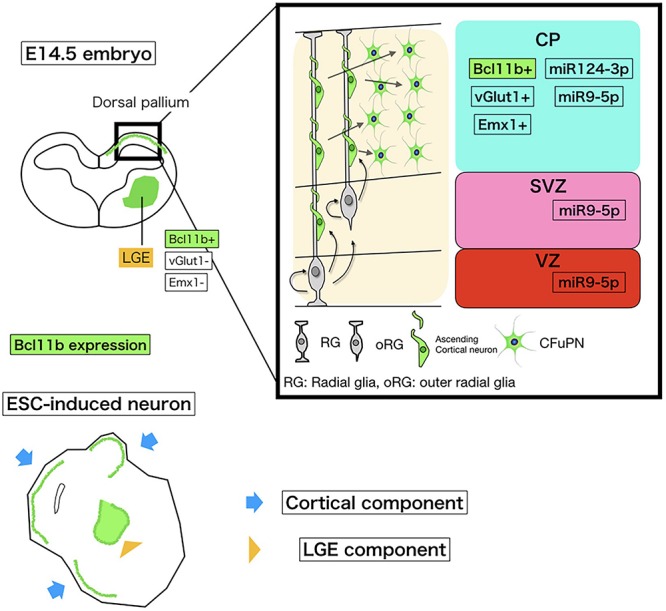
Schematic of miRNA and Bcl11b expression in embryonic corticogenesis and induced neurons. The upper left schema is a coronal section of the embryonic brain at E14.5. The bold square shows the dorsal pallium growing to the cerebral cortex. Around this stage, Bcl11b is expressed in CFuPNs in the dorsal pallium and lateral geniculate eminence (LGE). To collect CFuPNs by using Bcl11b expression as a marker, we sampled only the dorsal pallium for microarray. The upper right shows Bcl11b and miRNA expressions in the dorsal pallium based on our array study and previous reports. CP, cortical plate; SP, subplate; SVZ, subventricular zone; and VZ, ventricular zone. CFuPNs in the CP express Bcl11b and miRNA124-3p. miRNA9-5p is expressed in all dorsal pallium cells. The lower cartoon shows an ESC-induced neuron. The ESC-induced neural sphere has a cortical component and LGG component, which both express Bcl11b. To discriminate Bcl11b + CFuPNs from LGE Bcl11b+ neurons, vGlut1, and Emx1 expression is also observed.

Our microarray data of transcription factor and miRNA expression indicated that miRNA-124-3p is exclusively expressed in the CFuPNs in embrio. (A schematic of our microarray data is shown in Figure 5.)

miRNA-124-3p regulates cell fate toward neurons (Makeyev et al., 2007; Yoo et al., 2009; Xue et al., 2013) with specification toward cortical neurons (Maiorano and Mallamaci, 2009; Akerblom et al., 2012; Barca et al., 2014; Sun et al., 2015; Åkerblom et al., 2015). Further, locked nucleic acid (LNA)-oligo *in situ* hybridization experiments showed that miRNA-124 is first expressed at E12.5 in postmitotic neurons in the cortical plate, but by E14.5 miRNA-124 expression shows a triphasic pattern: strong expression in the cortical plate, intermediate expression in the sub-ventricular zone, and no expression in the ventricular zone (Maiorano and Mallamaci, 2009; Nowakowski et al., 2011; Mao et al., 2014). Importantly, the distribution of Bcl11b in corticogenesis is similar to that of miRNA-124+ cells at E12.5 – E14.5 (Maiorano and Mallamaci, 2009; Nowakowski et al., 2011; Mao et al., 2014), supporting our result that CFuPNs are best separated by miRNA-124-3p.

Previous reports such as Miki et al. (2015) indicate that applying the miRNA switch to just one miRNA is sufficient to exceed the purification efficiency of multiple cell types compared to standard surface marker methods. However, it remains to be cleared whether this higher efficiency is sufficient for clinical translation. Because miRNA switches can be designed orthogonally, searching for other miRNA that act as markers for CFuPNs and preparing the appropriate miRNA switch would allow us to purify CFuPNs at even higher numbers than reported here.

In conclusion, miRNA-124-3p are specifically increased in CFuPN in corticogenesis and microRNA-responsive modified mRNA system of miRNA-124-3p enriched CFuPN-like cell from PSC-derived neuron.

## Data Availability Statement

The datasets generated for this study can be found in the NCBI Gene Expression Omnibus, GSE135924, https://www.ncbi.nlm.nih.gov/geo/query/acc.cgi?acc=GSE135924.

## Ethics Statement

The animal study was reviewed and approved by the Institutional Animal Care and Use Committee of Animal Research Facility, CiRA, Kyoto University (Permission Number: 10-8-9).

## Author Contributions

TS, AM, SMi, HS, and JT designed the research. TS, AM, and SMa performed the research. TS, AM, SMa, and HS analyzed the data. TS, AM, and JT wrote the manuscript.

## Conflict of Interest

The authors declare that the research was conducted in the absence of any commercial or financial relationships that could be construed as a potential conflict of interest.

## Supplementary Material

FIGURE S1 Composition of the Bcl11b/IRES-EGFP targeting vector. Schema of the BAC-recombination technique using RP23-351K8 and two recombination steps to create the targeting vector, Bcl11b/IRES-EGFP-PGK-EM7-Bsd.

FIGURE S2 Separation of CFuPNs by GFP expression with FACS from the dorsal pallium of Bcl11b-IRES-EGFP knock-in mice at E16.5. **(A)** Histogram of GFP-positive (green) and GFP-negative (blue) cells separated by FACS at E16.5. **(B)** The percentage of positively gated and negatively gated cells. **(C)** qPCR analysis shows CFuPN-specific gene expressions were increased in the GFP-positive population. Student’s *t*-test. ^∗∗^*p* < 0.0001. *n* = 3.

FIGURE S3 Induction of mouse ESC-derived cortical neurons. **(A)** The induction protocol. **(B–G)** Immunocytochemistry of sliced spheroids of mouse ESC-derived cortical neurons at day 15. Cells forming rosettes expressed the telencephalic markers Foxg1 and Lhx2 **(B,E)**. Each rosette was lined with Bcl11b-positive projection neurons **(C,F)** that were also positive for Tuj1 and Emx1 **(D,G)**, indicating mature neuronal and dorsal pallium character. Scale bars, 40 μm **(B,E)** and 20 μm **(C,D,F,G)**. **(H)** qPCR of cortical neuron-specific markers. Error bars, SD (*n* = 3).

FIGURE S4 Enrichment of CFuPN-like cells by miRNA switches. **(A)** Cell sorting of Bcl11b-EGFP knock-in mouse ESC-derived cortical neurons by miRNA 124-3p, 9-5p, and 219-5p switches. **(B)** The purity of GFP-positive cells in each fraction were 63.00 ± 6.86, 41.80 ± 17.55, and 33.20 ± 5.92% for miRNA124-3p, miRNA9-5p and miRNA219-5p switches, respectively (*n* = 3).

FIGURE S5 qPCR analysis for separated neurons by miRNA 124-3p, 9-5p, and 219-5p switches. qPCR data for projection neuron markers. Bcl11b and Ldb2, the marker for sub-cerebral projection neuron were enriched by miRNA-124-3p switch, however, Satb2, a callosal projection neuron marker or Tbr1, a corticothalamic projection marker were not (Student’s *t*-test. ^∗∗^*p* < 0.01. *n* = 3).
