# Supplementary figures and images for: MicroRNA-Based Separation of Cortico-Fugal Projection Neuron-Like Cells Derived From Embryonic Stem Cells
Source: Front Neurosci. 2019 Oct 23;13:1141. doi: 10.3389/fnins.2019.01141 (PMC6819314; doi:10.3389/fnins.2019.01141)

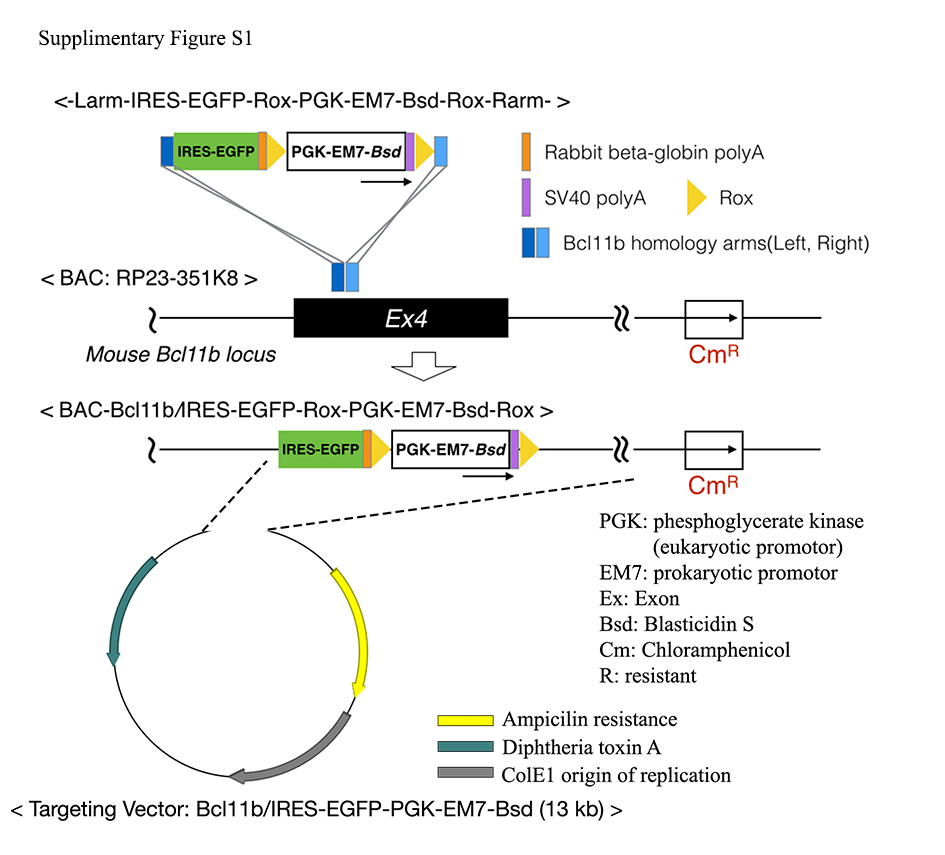

Supplement: FIGURE S1 — Composition of the Bcl11b/IRES-EGFP targeting vector. Schema of the BAC-recombination technique using RP23-351K8 and two recombination steps to create the targeting vector, Bcl11b/IRES-EGFP-PGK-EM7-Bsd. [file Image_1.TIF]

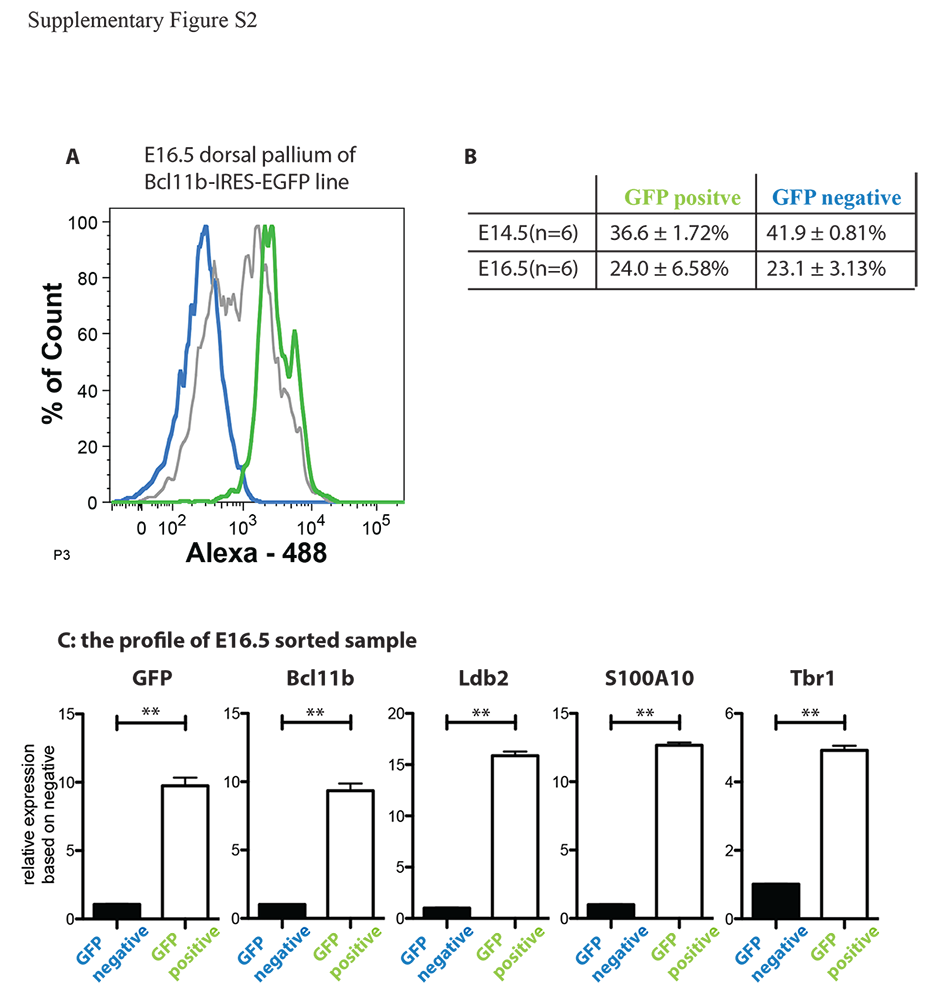

Supplement: FIGURE S2 — Separation of CFuPNs by GFP expression with FACS from the dorsal pallium of Bcl11b-IRES-EGFP knock-in mice at E16.5. (A) Histogram of GFP-positive (green) and GFP-negative (blue) cells separated by FACS at E16.5. (B) The percentage of positively gated and negatively gated cells. (C) qPCR analysis shows CFuPN-specific gene expressions were increased in the GFP-positive population. Student’s t-test. ∗∗p < 0.0001. n = 3. [file Image_2.TIF]

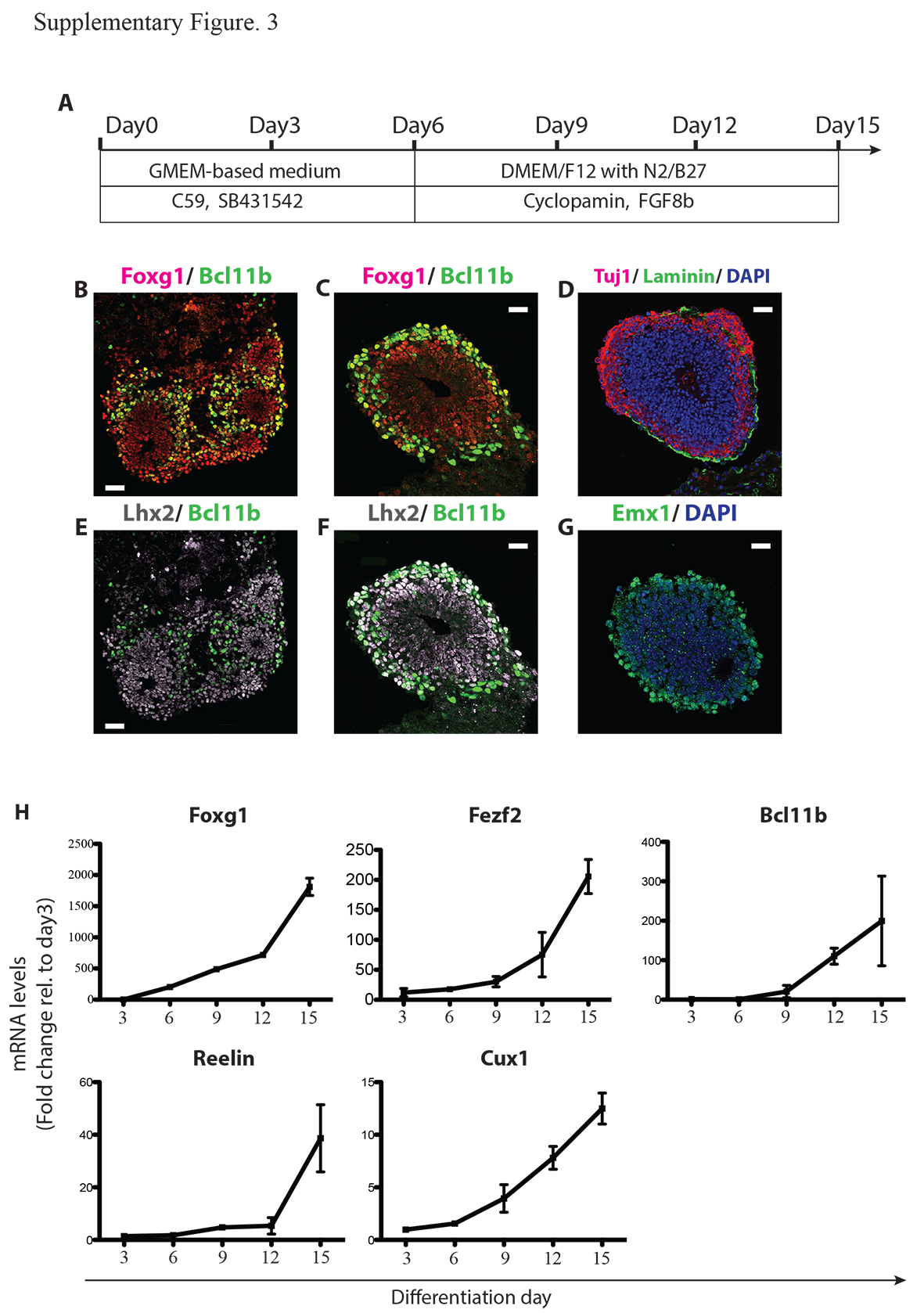

Supplement: FIGURE S3 — Induction of mouse ESC-derived cortical neurons. (A) The induction protocol. (B–G) Immunocytochemistry of sliced spheroids of mouse ESC-derived cortical neurons at day 15. Cells forming rosettes expressed the telencephalic markers Foxg1 and Lhx2 (B,E). Each rosette was lined with Bcl11b-positive projection neurons (C,F) that were also positive for Tuj1 and Emx1 (D,G), indicating mature neuronal and dorsal pallium character. Scale bars, 40 μm (B,E) and 20 μm (C,D,F,G). (H) qPCR of cortical neuron-specific markers. Error bars, SD (n = 3). [file Image_3.TIF]

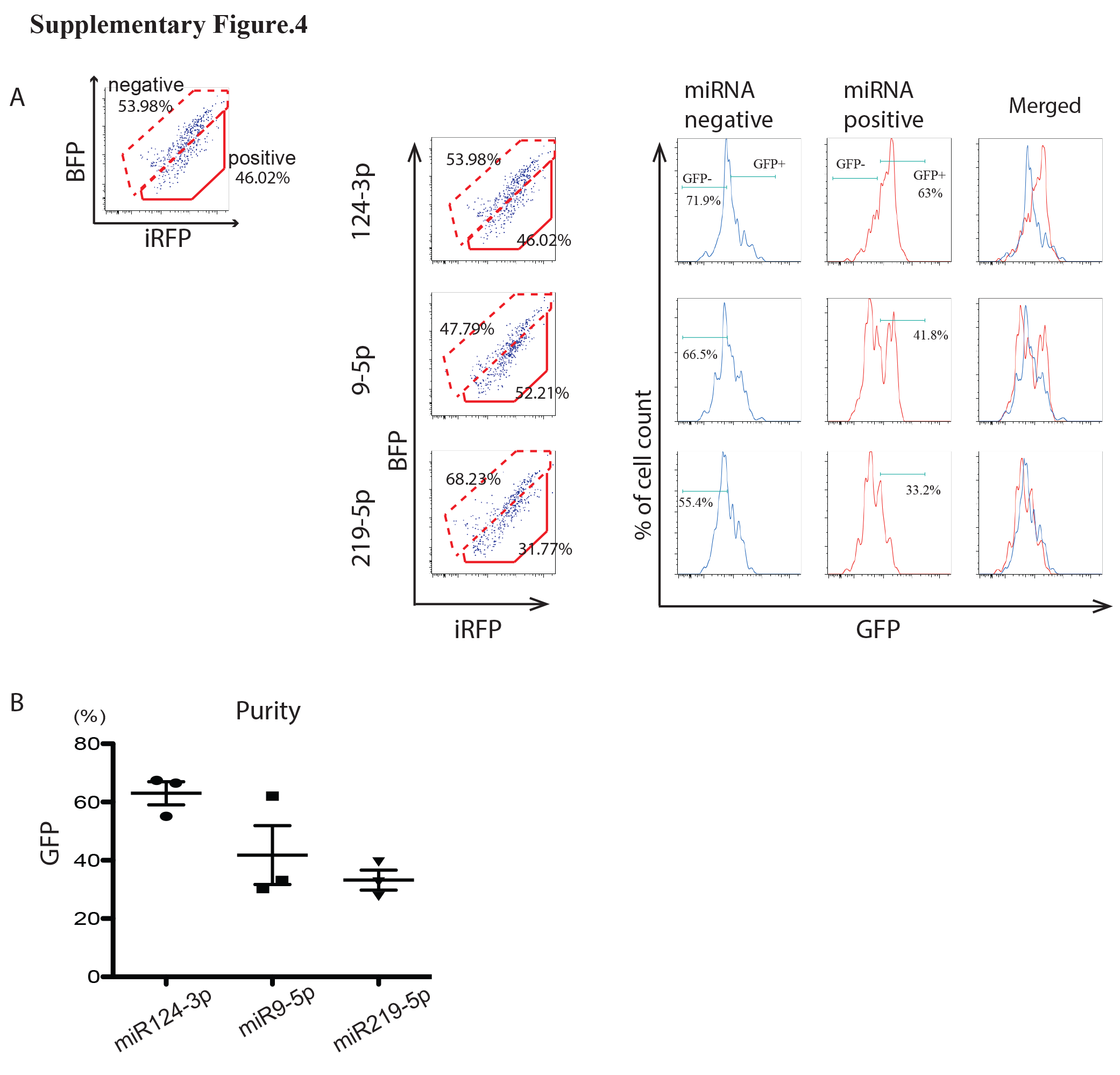

Supplement: FIGURE S4 — Enrichment of CFuPN-like cells by miRNA switches. (A) Cell sorting of Bcl11b-EGFP knock-in mouse ESC-derived cortical neurons by miRNA 124-3p, 9-5p, and 219-5p switches. (B) The purity of GFP-positive cells in each fraction were 63.00 ± 6.86, 41.80 ± 17.55, and 33.20 ± 5.92% for miRNA124-3p, miRNA9-5p and miRNA219-5p switches, respectively (n = 3). [file Image_4.TIF]

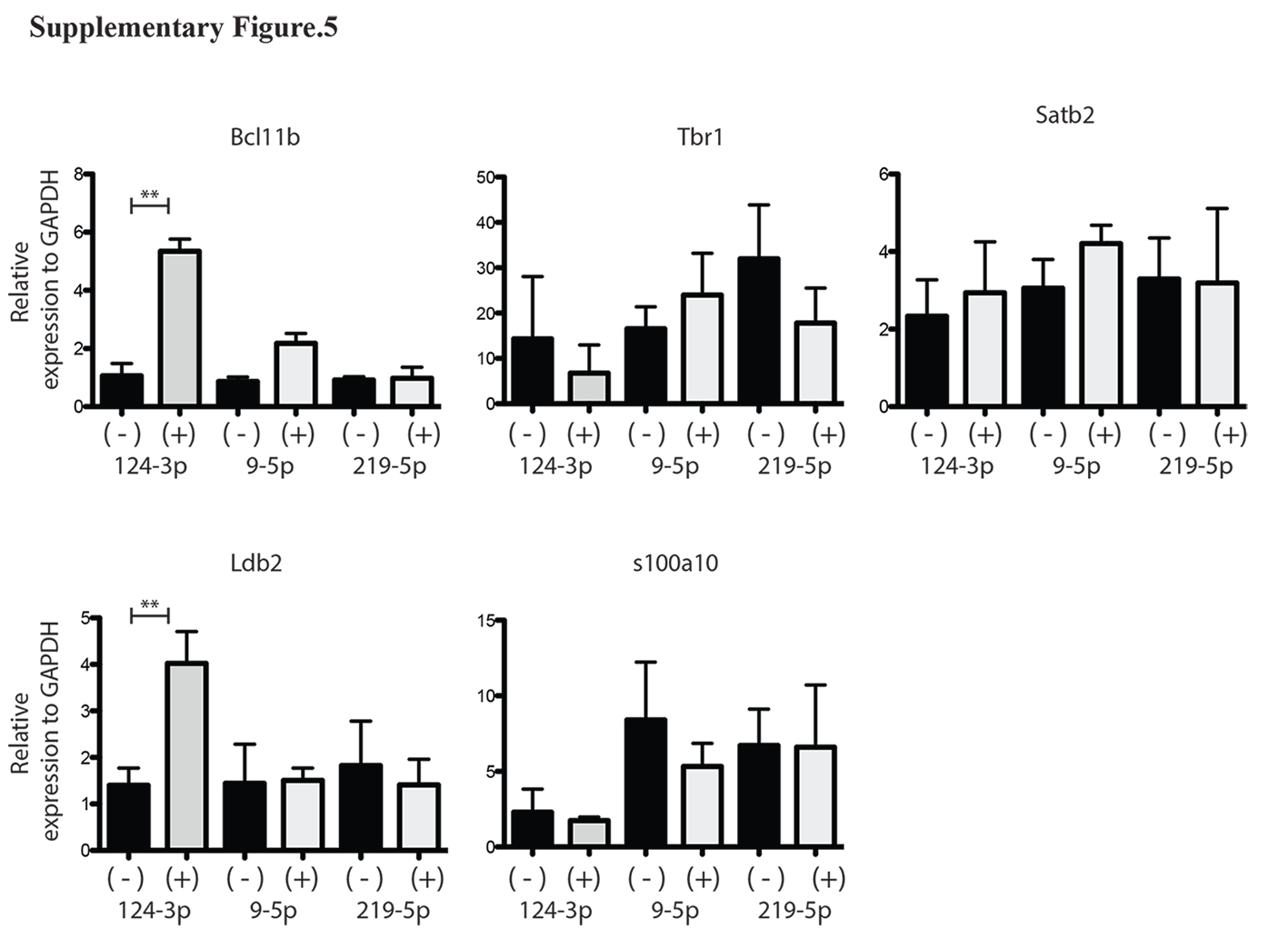

Supplement: FIGURE S5 — qPCR analysis for separated neurons by miRNA 124-3p, 9-5p, and 219-5p switches. qPCR data for projection neuron markers. Bcl11b and Ldb2, the marker for sub-cerebral projection neuron were enriched by miRNA-124-3p switch, however, Satb2, a callosal projection neuron marker or Tbr1, a corticothalamic projection marker were not (Student’s t-test. ∗∗p < 0.01. n = 3). [file Image_5.tif]
